# Risk Stratification of Latent Tuberculosis Defined by Combined Interferon Gamma Release Assays

**DOI:** 10.1371/journal.pone.0043285

**Published:** 2012-08-17

**Authors:** Véronique Corbière, Gaelle Pottier, Florence Bonkain, Kinda Schepers, Virginie Verscheure, Sophie Lecher, T. Mark Doherty, Camille Locht, Françoise Mascart

**Affiliations:** 1 Laboratory of Vaccinology and Mucosal Immunity, Université Libre de Bruxelles (U.L.B.) Brussels, Belgium; 2 Department of Nephrology, Hôpital Erasme, Université Libre de Bruxelles (U.L.B.) Brussels, Belgium; 3 Immunodeficiency Unit, Hôpital Erasme, Université Libre de Bruxelles (U.L.B.) Brussels, Belgium; 4 INSERM, U1019, Lille, France; 5 CNRS, UMR8204, Lille, France; 6 Université de Lille Nord de France, Institut Pasteur de Lille, Lille, France; 7 Center for Infection and Immunity of Lille, Lille, France; 8 Department of Tuberculosis Immunology, Statens Serum Institute, Copenhagen, Denmark; 9 Immunobiology Clinic, Hôpital Erasme, Université Libre de Bruxelles (U.L.B.), Brussels, Belgium; University of Palermo, Italy

## Abstract

**Background:**

Most individuals infected with *Mycobacterium tuberculosis* develop latent tuberculosis infection (LTBI). Some may progress to active disease and would benefit from preventive treatment yet no means currently exists to predict who will reactivate. Here, we provide an approach to stratify LTBI based on IFN-γ responses to two antigens, the recombinant Early-Secreted Antigen Target-6 (rESAT-6) and the latency antigen Heparin-Binding Haemagglutinin (HBHA).

**Methods:**

We retrospectively analyzed results from in-house IFN-γ-release assays with HBHA (HBHA-IGRA) and rESAT-6 (rESAT-6-IGRA) performed during a 12-year period on serial blood samples (3 to 9) collected from 23 LTBI subjects in a low-TB incidence country. Both the kinetics of the absolute IFN-γ concentrations secreted in response to each antigen and the dynamics of HBHA/rESAT-6-induced IFN-γ concentrations ratios were examined.

**Results:**

This analysis allowed the identification among the LTBI subjects of three major groups. Group A featured stable HBHA and rESAT-6-IGRA profiles with an HBHA/rESAT-6 ratio persistently higher than 1, and with high HBHA- and usually negative rESAT-6-IGRA responses throughout the study. Group B had changing HBHA/rESAT-6 ratios fluctuating from 0.0001 to 10,000, with both HBHA and rESAT-6 responses varying over time at least once during the follow-up. Group C was characterized by a progressive disappearance of all responses.

**Conclusions:**

By combining the measures of IFN-γ concentrations secreted in response to an early and a latency antigens, LTBI subjects can be stratified into different risk groups. We propose that disappearing responses indicate cure, that persistent responses to HBHA with HBHA/rESAT-6 ratios ≥1 represent stable LTBI subjects, whereas subjects with ratios varying from >1 to <1 should be closely monitored as they may represent the highest-risk group, as illustrated by a case report, and should therefore be prioritized for preventive treatment.

## Introduction

Despite predictions of a decline in the global incidence of tuberculosis (TB), the number of new cases continues to increase, with nearly 10 million new cases in 2010 [1]. In addition to clinical TB, more than 2 billion individuals are thought to have latent *Mycobacterium tuberculosis* (*Mtb*) infection (LTBI). Although they show no sign of disease, they are at risk of reactivating the infection, and approximately 10% of them will eventually develop the disease [2] and would therefore benefit from isoniazid preventive treatment (IPT) [3]. However, IPT is not without adverse effects and, due to the enormous number of LTBI subjects, it would be impractical to treat all of them. A more realistic approach is to preventively treat only those who are at the highest risk of reactivation. It is well established that acquired or induced immuno-suppression significantly augments the risk of reactivation [4]–[9]. However, this accounts only for a small fraction of LTBI subjects progressing to active disease, and most reactivation TB patients suffer from no obvious underlying immunodeficiency.

LTBI represents a wide spectrum running from the eventual elimination of the bacilli to subclinical disease [2], [10], which may be a reflection of changes in *Mtb* gene expression profiles in response to the different micro-environmental conditions encountered by the bacilli within the infected host. These various expression profiles would be expected to result in differences in immune responses of the host [10], and by identifying these differences, the identification of different stages of LTBI should become feasible.

Commercially available IFN-γ release assays (IGRAs) measuring the IFN-γ concentrations released after a 24 hrs *in vitro* stimulation of whole blood with peptides from antigens encoded in the RD-1 region of the *Mtb* genome can identify LTBI subjects [11], but they were reported to lack sensitivity [12]–[14], and they provide no clue as to the risk for reactivation TB [15], [16]. They also cannot differentiate active from latent TB, unless they are combined with the analysis of the IFN-γ response to another antigen, such as a recombinant and methylated form of the Heparin-Binding Haemagglutinin (HBHA) [17].

We have recently developed a 96 hrs peripheral blood mononuclear cell (PBMC) IGRA using the native form of the latency antigen HBHA and being a very sensitive and specific assay to detect all LTBI subjects and to discriminate them from patients with active TB [12]. No interference of a past BCG vaccination was noted, and this HBHA-IGRA was more sensitive than the commercial IGRAs for the detection of LTBI [12]. It was also by far more sensitive than an IGRA using recombinant Early-Secreted Antigen Target-6 (rESAT-6) to detect LTBI [12], indicating heterogeneity among LTBI subjects who may produce IFN-γ in response to both antigens or to only to one of them. This heterogeneity may be expressed for each subject by a ratio between the HBHA- and rESAT-6-induced IFN-γ concentrations. A retrospective analysis of the results of these HBHA and rESAT-6-IGRAs for up to 12 years for 23 subjects with well-defined LTBI, has allowed us to characterize the evolution of the HBHA/rESAT-6-induced IFN-γ ratios over time. These ratios were remarkably stable for some subjects and highly variable for others. Based on the dynamics of these ratios, we provide here a first attempt to classify the LTBI subjects in different risk categories for the development of active TB. The proposed model is substantiated by a case report illustrating the potential clinical value of this approach.

## Materials and Methods

### Latently-infected Subjects

From 2000 to 2008, twenty-three LTBI subjects were recruited among hospital health care workers (HCW) in Belgium where the study was performed and followed up to 2011. Their main demographic and clinical characteristics are given in Table 1, comprising the BCG vaccination status, the induration diameter size of the tuberculin skin test (TST) at the time of its conversion, the duration of the follow-up since the TST conversion, and the results of the QuantiFERON-TB Gold in-Tube (QFT-G-IT). In Belgium every one to two years TST are performed in HCW to detect a potential new *Mtb* infection by injection of 2 tuberculin units of Purified Protein Derivative (PPD, RT23, Statens Serum Institute, SSI, Copenhagen, Denmark), and reading 48 to 72 hrs later. LTBI were defined according to national guidelines (www.fares.be) by TST conversion (increase in the induration diameter size of ≥10 mm compared to the previous TST), normal chest radiography, and no clinical sign of disease, thereby fulfilling strict criteria for LTBI. The QFT-G-IT was performed as recommended by the supplier (Cellestis, Melbourne, Australia) since 2006 for comparison purposes with the other tests reported here, but was not used for the clinical evaluation of HCW for their potential LTBI status.

**Table 1:** Characteristics of the LTBI subjects included in the study.

Subjectnumber	Age*(years)	Sex	EthnicOrigin	BCG status	Year of TST conversion	Induration diameter (mm)	Time since TST conversion (years)	QTF-G-IT	Follow-up period (years)	Treatment	Possible *Mtb*re-exposure
1	55	F	WE	+	1994	20	12	–	5	–	no
2	39	F	NA	+	1992	40	8	–	11	–	yes (T)
3	24	M	SA	–	2001	18	0	–	9	–	no
4	42	F	WE	+	1993	10	9	–	9	–	no
5	57	F	WE	+	1996	17	10	–	5	+	no
6	25	M	WE	+	2000	18	2	–	9	+	no
7	27	F	CA	+	2003	N/A	5	+	4	–	no
8	27	F	WE	–	1986	N/A	15	+/−^#^	9	–	no
9	40	F	NA	+	1995	22	5	–	11	+	yes (T)
10	35	F	CA	+	2008	10	0	–	2	–	yes (T)
11	43	M	NA	+	1989	30	17	+	5	–	yes (T)
12	48	F	WE	+	1994	18	14	++/−§	4	+	no
13	32	F	NA	–	2000	16	0	++	12	–	yes (T)
14	29	F	WE	+	2000	20	1	++	9	+	yes (W)
15	45	F	WE	N/A	2006	11	2	++/+°	4	–	no
16	41	M	WE	–	1972	10	29	++/−°	11	–	yes (T)
17	42	M	WE	+	1984	12	16	–	11	–	no
18	49	M	NA	+	1978	N/A	28	+	3	–	yes (T)
19	43	F	CA	+	2007	14	1	–	4	–	no
20	50	F	WE	+	1985	25	17	–	8	+	yes (W)
21	43	F	WE	–	1998	15	4	–	8	+	no
22	45	F	WE	+	1990	20	16	–	6	+	no
23	39	F	WE	+	1986	30	15	–	10	–	no

**Footnotes:** *at enrolment, F: Female, M: Male, Ethnic Origin: WE Western Europe, NA North Africa, SA South Africa, CA Central Africa, BCG: Bacille Calmette et Guerin, N/A: Not Applicable, TST: Tuberculin Skin Test, QFT-G-IT score results:

−0–0.34 IU/ml;

+0.35–5 IU/ml

++>5 IU/ml;

#conversion;

§ reversion;

°fluctuations of the score results, *Mtb*: Mycobacterium tuberculosis, T: subjects travelling for visiting friends or relatives in TB-high-endemic countries, W: subjects working in at risk department.

HBHA and rESAT-6-IGRAs in response to the different mycobacterial antigens were performed at several time points after enrolment, with, however, some data missing due to limited blood samples or antigen availability.

### Ethics Statement

The study was approved in 2000 by the ethical committee of our university, the “Ethical Committee of the Université Libre de Bruxelles”, and verbal consent was required. In 2007, following administrative changes, our laboratory became dependent on another ethics committee, “the Ethics Committee of Erasme Hospital” (N°OMO21). Our study was resubmitted, and, in response to the growing exigency of human research ethics, written consent was thereafter required from all participants. During follow-up, patients enrolled before 2007 were asked to confirm their verbal consent with a written one. We therefore have the written consent forms of all participants.

### IGRA in Response to HBHA and rESAT-6

The HBHA and rESAT-6-IGRAs used in this study were previously standardized to detect LTBI among HCW and to discriminate them from non-infected subjects [14]. Briefly, PBMC were purified from fresh blood samples, and 10^6^ PBMC were stimulated with 2 µg/ml HBHA or 10 µg/ml rESAT-6, as previously described [12]. HBHA was purified from *Mycobacterium bovis* BCG as detailed elsewhere [18], [19]. Recombinant ESAT-6 was obtained from the Statens Serum Institute (SSI, Copenhagen, Denmark) and was available only since 2005. After 96-hrs, the IFN-γ concentrations released in the supernatants were measured by ELISA [20] and the IFN-γ secretion from unstimulated PBMC was subtracted from the antigen-induced IFN-γ secretion. Stimulation with 2 µg/ml phytohemagglutinin A (HA16, Murex Biotech Ltd, Dartford, UK) served as a positive control of cellular immune responses and stimulation with 4 µg/ml PPD (SSI) was used as a control of immune responses to mycobacterial antigens. The sensitivity of the IFN-γ ELISA was 10 pg/ml. The cut-off values for optimal discrimination between non-infected controls and LTBI subjects were previously determined: 100 pg/ml for HBHA, 40 pg/ml for rESAT-6 [12].

### Statistics

GraphPad Prism version 4.03 for Windows (GraphPad Software, San Diego, CA, USA, www.graphpad.com) was used for statistical analyses. Comparisons of continuous variables between groups were performed with the non-parametric Kruskal-Wallis test combined with the Dunn’s multiple comparison tests. Fisher’s test was used to determine whether classification of LTBI subjects was biased by the ethnic origin, BCG vaccination, IPT and re-exposure risk. Values of p<0.05 were considered to be significant.

## Results

### Dynamic of the HBHA- and rESAT-6-IGRA Results

**Figure 1:**
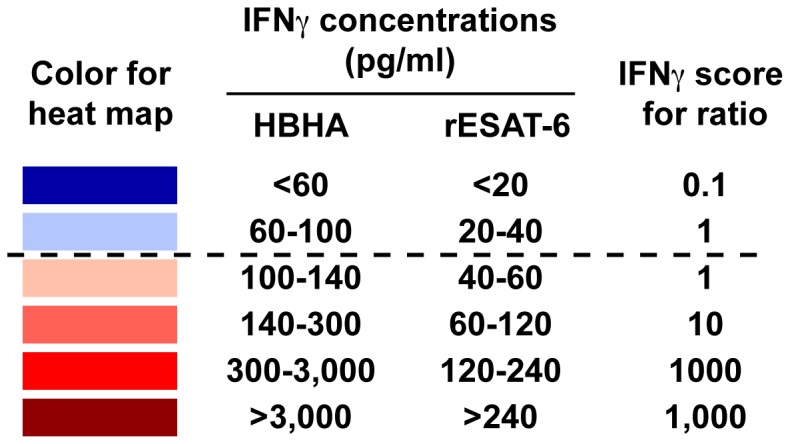
Definition of the heat map color and of the score values for HBHA- and rESAT-6-IGRA. The first column shows the intensity of the colors used in the heat map to represent the intensity of the IFN-γ responses, from dark blue for insignificant IFN-γ responses to dark red for very high responses. The last column defines the IFN-γ scores used to calculate the HBHA/rESAT-6 ratios. These scores were attributed to the different ranges of IFN-γ concentrations illustrated in the heat map. Values corresponding to the grey zone just below or above the cut-off values (dotted line), were attributed the same score of 1.

**Figure 2:**
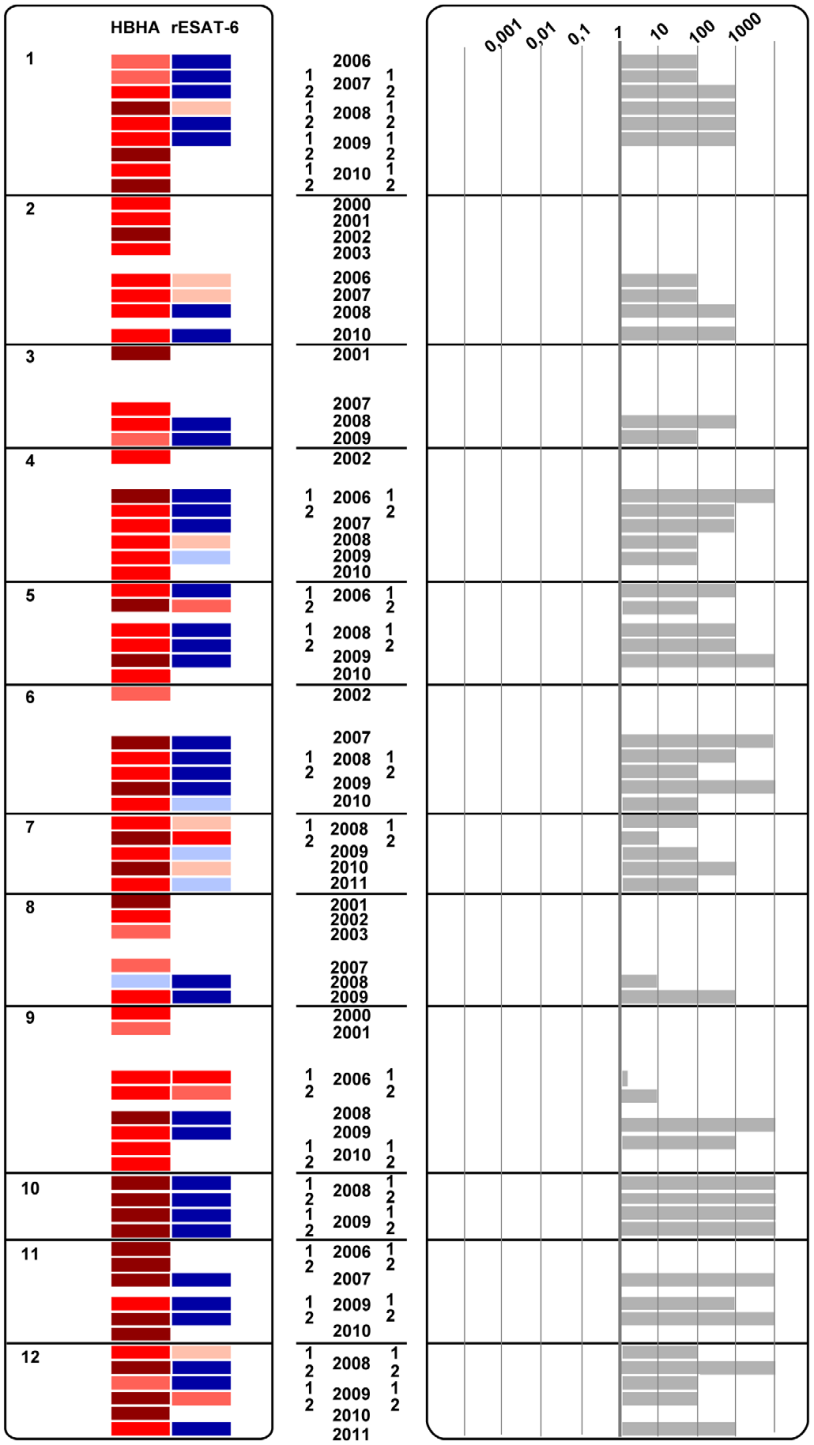
LTBI subjects displaying stable HBHA- and rESAT-6-IGRA profiles to mycobacterial antigens over time (group A). HBHA- and rESAT-6-IGRAs were performed on 12 LTBI subjects, numbered from 1 to 12, at different time points between the years 2000 and 2011, as indicated. When indicated, the numbers 1 and 2 correspond to tests performed twice, i. e. in the first and second part of the year, respectively. A heat-map is represented on the left panel, with positive HBHA- and rESAT-6-IGRA results shown in red and negative results shown in blue as defined in Figure 1. The right panel represents ratios between HBHA- and rESAT-6-induced IFN-γ concentrations. White boxes correspond to time points for which no data was available for the particular antigen.

**Figure 3:**
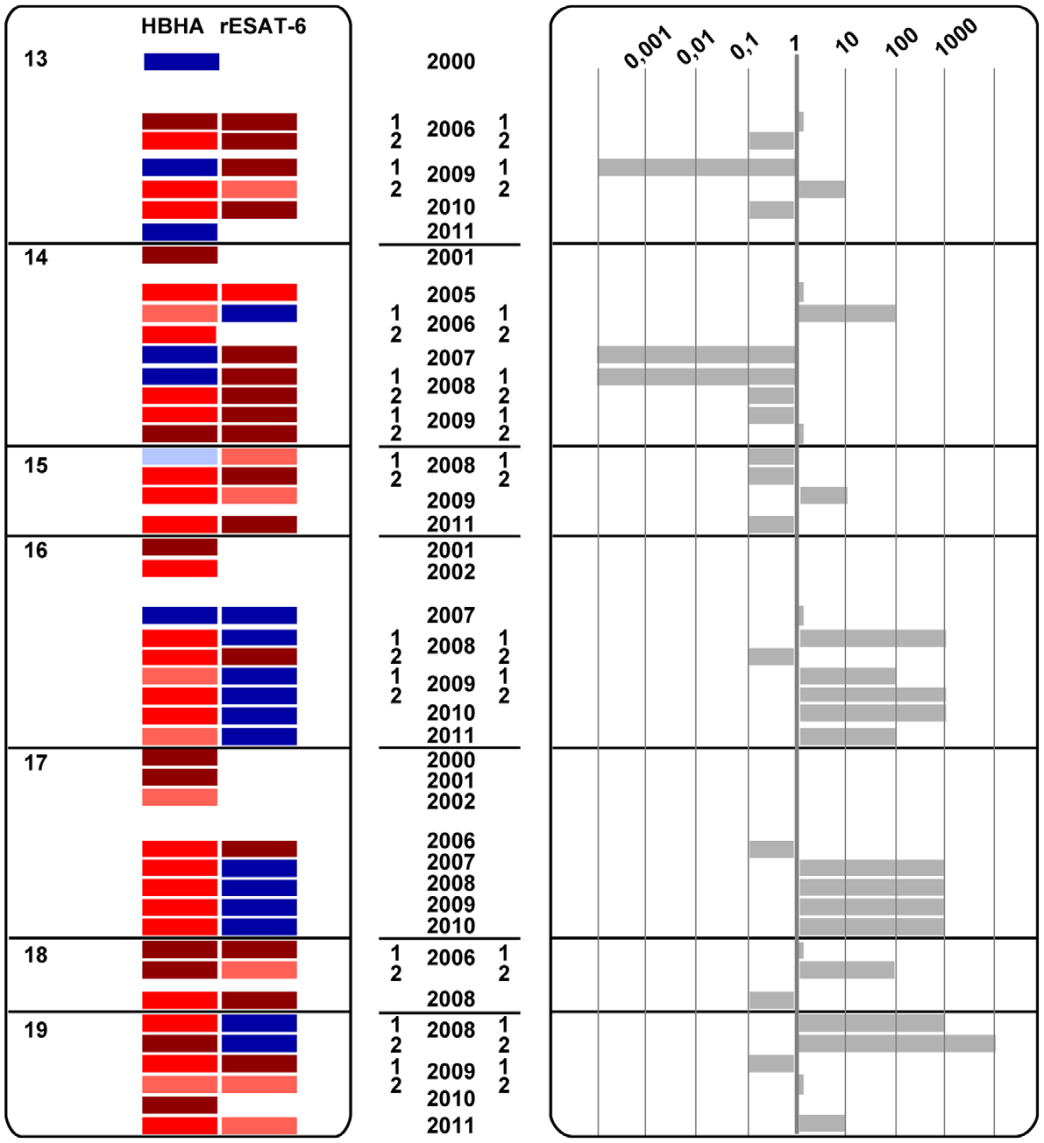
LTBI subjects displaying unstable HBHA- and rESAT-6-IGRA profiles to mycobacterial antigens over time (group B). HBHA- and rESAT-6-IGRAs were performed on 7 LTBI subjects, numbered from 13 to 19, at different time points between the years 2000 and 2011, as indicated. Heat-map illustrating the intensity of IFN-γ responses over time, and ratios between HBHA- and rESAT-6-induced IFN-γ concentrations are represented in panel A and B, respectively. When indicated, the numbers 1 and 2 correspond to tests performed twice, i. e. in the first and second part of the year, respectively. White boxes correspond to time points for which no data was available for the particular antigen.

**Figure 4:**
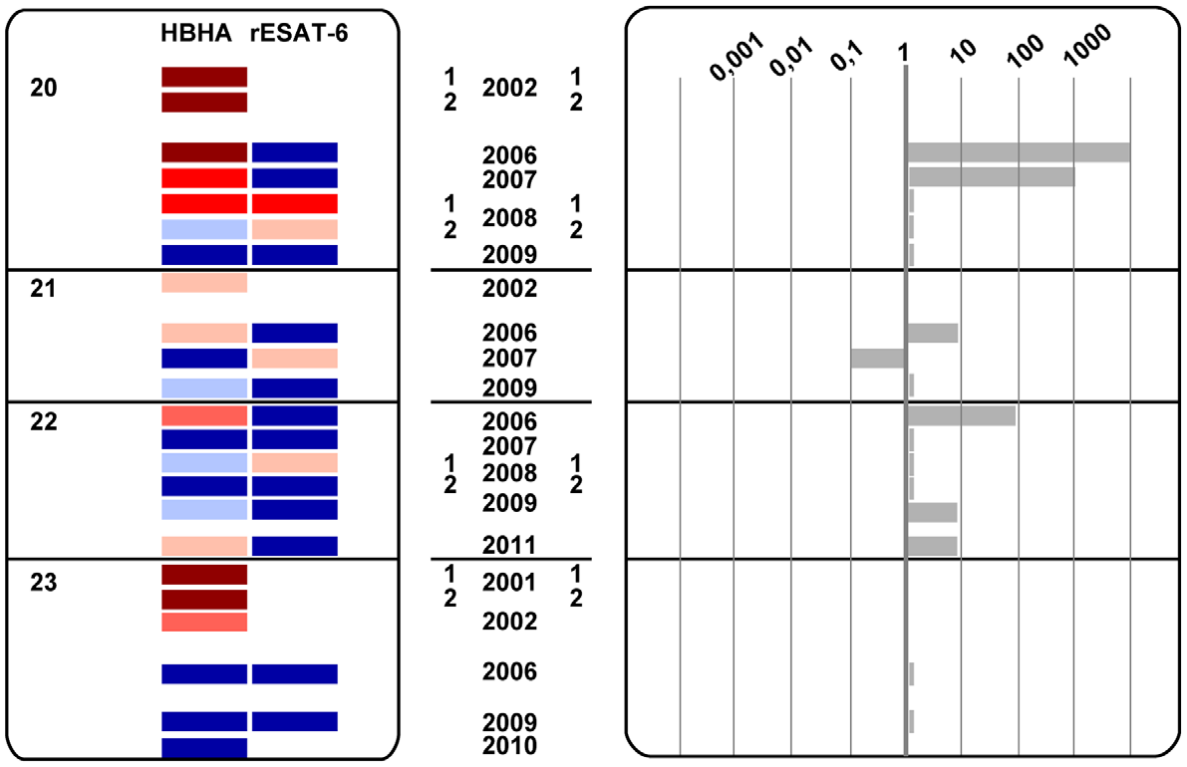
LTBI subjects displaying disappearing HBHA- and rESAT-6-IGRA responses to mycobacterial antigens over time (group C). HBHA- and rESAT-6-IGRAs were performed on 4 LTBI subjects, numbered from 20 to 23, at different time points between the years 2001 and 2011, as indicated. Heat-map illustrating the intensity of IFN-γ responses over time, and ratios between HBHA- and rESAT-6-induced IFN-γ concentrations are represented in panel A and B, respectively. When indicated, the numbers 1 and 2 correspond to tests performed twice, i. e. in the first and second part of the year, respectively. White boxes correspond to time points for which no data was available for the particular antigen.

The dynamics of the IFN-γ concentrations released in response to HBHA for up to 12 years and for rESAT-6 for the last 6 years are visually represented by heat-maps. A scale from light red to dark red and from light blue to dark blue depicts the low to strong positive and the borderline to clearly negative values, respectively, as indicated in figure 1. Considering a classical coefficient of variation (CV) of 20% for the ELISA, “grey zones” were defined as +/−2 CV around the cut-off value, and are represented by the light blue or very light red colors, representing values below and above the cut-off values, respectively. Different patterns of evolution over time of the IFN-γ concentrations secreted in response to HBHA and to rESAT-6 clearly appear on figures 2,3,4 indicating that groups of subjects can be identified with some individuals most often responding only to HBHA (Figure 2, left panel), others responding sometime or always both to HBHA and to rESAT-6 (Figure 3, left panel), and with a third group characterized by disappearing responses to both HBHA and rESAT-6 (Figure 4, left panel).

To further define the classification criteria of the individuals, ratios between HBHA- and rESAT-6-induced IFN-γ concentrations were calculated, based on scores of IFN-γ concentrations attributed similarly to the colors in the heat map and defined in figure 1. However, the values corresponding to the grey zone were attributed the same score of 1, as small differences are probably only a consequence of the coefficient of variation.

The analysis of these ratios and of the heat maps illustrated on figures 2,3,4 has allowed us to identify three different groups of LTBI subjects, respectively those with stable ratios along the time, persistently higher than 1 (Figure 2, group A), those with changing ratios varying from above to below one and vice-versa (Figure 3, group B), and finally those with disappearing IFN-γ responses (Figure 4, group C).

### LTBI Subjects with a HBHA/rESAT-6-IGRA Ratio Persistently Higher than One

**Figure 5:**
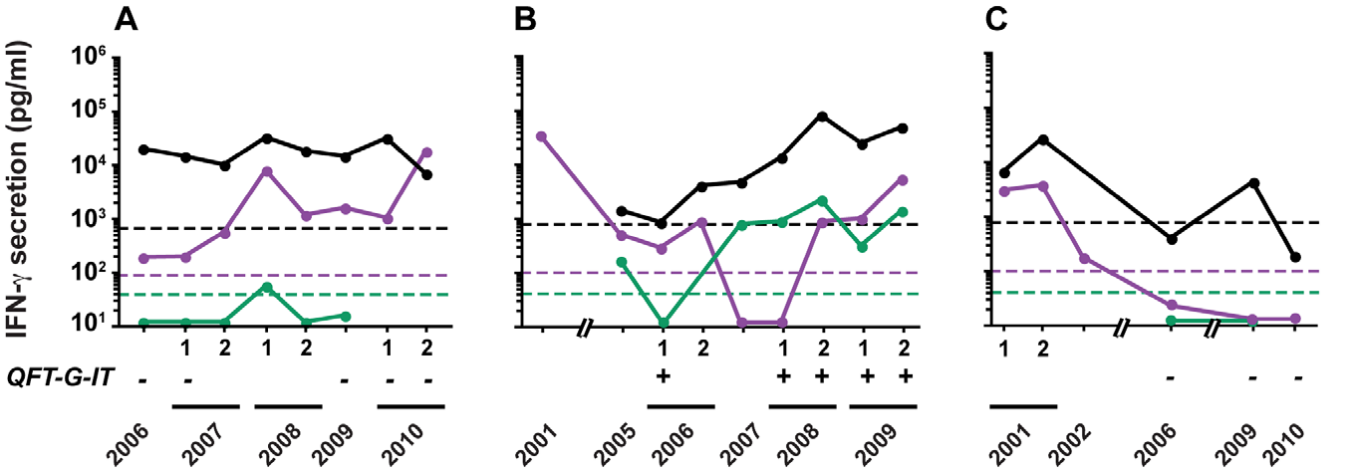
HBHA- and rESAT-6-IGRA kinetics of LTBI subjects representative of group A, B and C. IFN-γ secretion was measured by ELISA after four days of PBMC stimulation with PPD (black circle), HBHA (violet circle) or rESAT-6 (green circle) at the indicated time points. The limits of positivity for PPD, HBHA and rESAT-6 are represented as dotted lines with the corresponding color. When available, the negative or positive results of the QFT-G-IT are shown on the bottom of the figure as “−” or “+”, respectively. Subject shown on panel A belongs to group A (n°1) characterized by stable HBHA- and rESAT-6-IGRA responses. Subject shown on panel B belongs to group B (n°14) characterized by unstable HBHA- and rESAT-6-IGRA responses. Subject shown on panel C belongs to group C (n°23) presenting disappearing HBHA- and rESAT-6-IGRA responses.

Twelve out of the 23 LTBI subjects displayed a relatively stable HBHA- and rESAT-6-IGRA profile with HBHA/rESAT-6- IFN-γ ratios persistently ≥1, albeit varying between 1 and 10,000 (Figure 2, subjects 1–12 in Table 1). In this group, the HBHA-IGRAs were remarkably stable with IFN-γ concentrations fluctuating between high (red) and very high (dark red), with only one occasional value in the grey zone for subject n°8 (Figure 2, left panel). At the end of the study, a median of 2,113 pg/ml IFN-γ (IQ, 404–4,236) was obtained for the HBHA-IGRA, which is not significantly different from that obtained at enrolment. In contrast, the responses to rESAT-6, available since 2005, were most often persistently negative or in the grey zone, and when occasionally positive, the value for rESAT-6 was always lower than that obtained in response to HBHA (Figure 2, left panel). As a consequence, the ratios between the HBHA- and the rESAT-6-induced IFN-γ scores were always ≥1 in this group of LTBI subjects (Figure 2, right panel). A representative HBHA- and rESAT-6-IGRA profile for this group is shown in figure 5A (subject n°1), illustrating also the stable positive response to PPD tested as a positive control.

### LTBI Subjects with a HBHA/rESAT-6-IGRA Ratio Varying from Above to Below One and Vice-versa

Seven out of the 23 LTBI subjects had a changing HBHA- and rESAT-6-IGRA profile, with HBHA/rESAT-6-IGRA ratios varying from 0.0001 to 10,000 (Figure 3, subjects 13–19 in Table 1). Three subjects were most often characterized by several ratios below 1 as a consequence of the high IFN-γ response to rESAT-6, with low or absent IFN-γ response to HBHA, resulting in low to very low ratio (subjects 13, 14, 15). A ratio higher than 1 was occasionally noted when IFN-γ responses to rESAT-6 were transiently lower. The other four subjects had only transiently HBHA/rESAT-6 IFN-γ response ratios below 1, associated with a transient rise of the IFN-γ response to rESAT-6 (subjects 16 to 19). IFN-γ responses to both antigens disappeared transiently in one of these subjects (n°16).

Even though the HBHA-induced IFN-γ levels both at enrolment and at the end of the follow-up were not significantly different from those noted in group A, with median concentrations of 1,151 pg/ml IFN-γ (IQ, 163–1,741) at the end, for most subjects in this group, IFN-γ responses to HBHA were highly variable, fluctuating from highly positive to low positive/negative and then highly positive again (Figure 3). In this group, the rESAT-6-induced IFN-γ concentrations were at least at some time points and sometimes persistently very high. A representative HBHA- and rESAT-6-IGRA profile of this group (subject n°14) is given in figure 5B and indicates that the rESAT-6- and HBHA-IGRA values often showed a degree of reciprocity, with rising IFN-γ concentrations in response to rESAT-6 when the HBHA-induced IFN-γ concentrations were declining, and vice-versa. In contrast, PPD-induced IFN-γ concentrations remained persistently positive as illustrated on figure 5B. This group likely represents the highest risk category as illustrated by the case report below, describing the occurrence of active TB in a patient with impaired cellular immunity.

### LTBI Subjects with Disappearing HBHA- and rESAT-6-IGRA Responses

In subjects n°20–23, the HBHA-IGRA became progressively negative with no return to positive values (Figure 4, left panel). In three of them, the rESAT-6-IGRA fluctuated from negative to slightly positive and finally to totally negative values, whereas for one subject (n°23), only two values were obtained for rESAT-6-IGRA and they were both negative (Figure 4, left panel). When both HBHA- and rESAT-6-IGRAs became totally negative, the value of the ratio was 1 (Figure 4, right panel). However, when one HBHA- or rESAT-6-IGRA result was in the grey zone, this resulted in a ratio between 0.1 and 10 with no significant relevance (Figure 4), thereby pointing out the need to consider both the ratios for relative values and the heat maps for absolute values. One representative profile of this group is illustrated in figure 5C (subject n°23) showing that even the IFN-γ response to PPD progressively disappeared. This group likely represents subjects who have cleared the infection.

### Intergroup Comparisons of Major Clinical Parameters

Relevant clinical parameters were compared between the different groups identified, and no statistically significant differences were noted between the groups for these parameters (Table 2). However, none of the 4 subjects belonging to group C originated from a high TB incidence country, and only one of them was possibly re-exposed to *Mtb* by working in the pneumology department in the endoscopic pulmonary medicine ward. In contrast, around half of the subjects from the two other groups originated from a high TB endemic country, and 4/12 and 4/7 from groups A and B, respectively, were possibly re-exposed. In addition, even if not statistically different, the median time since TST conversion was only 2.0 years in group B compared to 8.5 and 15.5 in groups A and C respectively. Noteworthy, 5/7 subjects from group B had a positive QFT-G-IT test, stable over time for 3 of them, compared to 4/12 positive tests for subjects in group A (stable over time for 2 subjects), whereas none of the subjects in group C had a positive QFT-G-IT during the follow-up period. Finally, whereas 3/4 subjects from group C had received IPT, 4/12 and only 1/7 in groups A and B, respectively, had received IPT.

**Table 2:** Intergroup comparisons of the major clinical parameters.

	Group A	Group B	Group C
**Number of inclusion**	12	7	4
**Age at the time of recruitment (years)** a	39.3 (27.2–45.8)	42.3 (31.7–45.1)	43.8 (41.1–47.7)
**BCG vaccination** b	9 (75.0%)	4* (66,7%)	3 (75.0%)
**Time since BCG (years)** a	24.5 (15–30)	32.0 (14.5–45.9)	23.0**
**Origin from a high-TB prevalence country** b	6 (50%)	3 (43%)	0
**Possible ** ***Mtb*** ** re-exposure** b	4 (33%)	4 (57%)	1 (25%)
**Time since TST conversion** a	8.5 (3.5–13.0)	2.0 (1.0–28.0)	15.5 (9.5–16.5)
**Induration diameter (mm)** a	18.0 (13.5–26.0)	13.0 (10.5–18.0)	22.5 (17.5–27.5)
**QFT-G-IT** b	4 (33%)	5 (71%)	0
**Follow-up period (years)** a	7.0 (4.5–9.5)	9.0 (4.0–12.0)	8.0 (7.0–9.0)
**Treatment** b	4 (33%)	1 (14%)	3 (75%)

a Median (25th-75th interquartiles);

b numbers (percentages);

* out of 6 subjects instead of 7;

** no interquartile available as the number of subjects in group C was too small.

### Case Report

A 46-year-old man originating from Morocco, with a medical history of end-stage renal disease secondary to type 2 diabetes mellitus presented with chills during haemodialysis (HD) sessions on January 2011. The patient was on HD since 2007, and in 2009, he was included in a prospective study to evaluate the utility of HBHA-IGRA, compared to TST for the detection of LTBI in HD patients (Dessein et al, manuscript in preparation). The QFT-G-IT was highly positive (13.58 IU/ml) as were the PPD- and HBHA-IGRAs with respectively 18,972 pg/ml and 7,287 pg/ml IFN-γ. These results, especially the high IFN-γ response to the latency antigen HBHA, indicated that the patient was latently infected with *Mtb*. During a January 2011 HD session, the patient presented with chills and fever, but no abnormalities were noted upon physical examination, except a weight loss of 4 Kg over the previous weeks. Blood cultures remained negative. A chest radiography revealed no infiltrates, cavities or calcifications. The patient denied prior exposition to *Mtb*, but the QFT-G-IT was still positive, with values similar to those found two years earlier (14.36 IU/ml), whereas the HBHA-IGRA had become totally negative. However, depletion of the CD4^+^ regulatory T cells from the PBMC, performed as described previously [21] and based on CD25^high^ T cell depletion by positive immunomagnetic selection, resulted in the re-appearance of a low but positive IFN-γ response to HBHA stimulation (134 pg/ml). At that time, the IGRA values to rESAT-6 were very high (3,855 pg/ml) so that the HBHA/rESAT-6 ratio was 0.001. Since these results strongly suggested that the patient had progressed from latent *Mtb* infection to active TB disease, a PET Scan analysis was done demonstrating a strong metabolic captation of cervical and mediastinal lymph nodes. A jugular lymph node was surgically removed and revealed the presence of a granuloma with caseous necrosis. Anti-tuberculosis treatment was immediately initiated (Nicotibine, Rifadine, Tebrazid, Myambutol) and resulted in progressive clinical improvement. Thirty-two days later, the lymph node culture was positive for drug-sensitive *Mtb*.

## Discussion

The most likely outcome of an encounter with *Mtb* is the establishment of LTBI without any sign of active TB, which, due to its pauci-bacillary nature, can only indirectly be identified by the immunological sensitization to mycobacterial antigens. However, LTBI is heterogeneous, and its fate ranges from spontaneous cure, through stable and persistent asymptomatic infection to progression towards active disease [10]. This is likely to result from a combination of both the physiological adaptation ability of the bacilli to different micro-environmental conditions at different anatomical sites within the infected host [2], [22] and of the ability of the host’s immune system to control or even eliminate mycobacterial replication.

For more than 100 years, the TST was the only tool available to detect LTBI, until more recently, commercial IGRAs improved specificity and perhaps sensitivity in certain settings [11]. However, none of these methods allow us to stratify the estimated two billion LTBI subjects into different groups corresponding to the different phases of the LTBI spectrum, and they are thus of limited value to target IPT to those individuals that are at highest risk of developing active TB [15], [16], [23]. In this paper, we show that by combining IGRAs in response to two different mycobacterial antigens, the recombinant early antigen rESAT-6 and the latency antigen HBHA, it is possible to subdivide LTBI subjects into several groups. The LTBI subjects in this study all live in a low TB incidence country, and most of them are not regularly re-exposed to *Mtb*. Therefore, their infectious status is really that of LTBI, rather than that of a recent re-infection. By following their IFN-γ responses to HBHA and rESAT-6 over a prolonged period of time, we identified three major HBHA- and rESAT-6-IGRA profiles.

**Figure 6:**
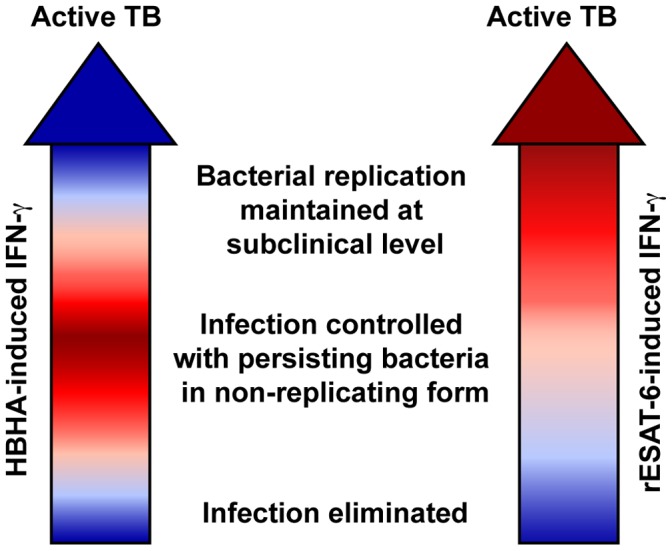
Spectrum of immune responses during LTBI. We propose to relate the spectrum of LTBI to the IFN-γ responses to two different mycobacterial antigens, HBHA represented in the left arrow, and rESAT-6 represented in the right arrow. The intensity of the colors depicts the intensity of IFN-γ responses with a scale described in Figure 1 (Adapted from Young et al. [10]).

Roughly half of the subjects displayed a stable HBHA- and rESAT-6-IGRA profile over up to 12 years, characterized by a ratio persistently ≥1 between the IFN-γ responses to HBHA and to rESAT-6. These subjects had mostly persistently high responses to HBHA and the responses to rESAT-6 were not detectable or had at each time point lower scores than the responses to HBHA. Since ESAT-6 is produced at high levels during the mycobacterial growth [24], this suggests that in these individuals growth of *Mtb* is being restricted. The persistently high response to HBHA suggests that the mycobacteria are still present and continue to produce the latency antigen, which is then presented to T cells. Interestingly, four of these subjects had undergone IPT, yet, their HBHA response did not disappear and remained actually quite high, suggesting that the treatment had not led to clearance of the bacilli. Alternatively, the sustained HBHA response may be due to the presence in peripheral blood of central memory T cells specific for the antigen [25]. However, the absence of a persistent response to other mycobacterial antigens, such as rESAT-6, as well as the observation that in some individuals the HBHA response disappears over time (group C, as discussed below) makes it unlikely that the persistence of central memory T cells are solely responsible for the sustained HBHA responses in this group. Although the interference of contacts with environmental mycobacteria cannot be formerly excluded, it is highly unlikely in this group of LTBI subjects defined by very strict criteria of TST conversion in HCW potentially exposed to *Mtb*-infected patients. In addition, a dissociation between the IFN-γ responses to latency antigens and to those encoded in the RD-1 region of the *Mtb* genome has been reported by other groups as well [14], [26]. Since HBHA is a protective antigen [27]–[30] and since in most LTBI subjects, in addition to IFN-γ secretion, the HBHA-specific T cells also express microbicidal and cytotoxic activities to mycobacteria-infected cells [31], we suggest that this group of LTBI subjects might be protected against TB re-activation and, unless undergoing immunosuppressive treatments, does not require IPT. However, large prospective studies will need to be performed to confirm this suggestion.

The second major group featured changing HBHA- and rESAT-6-IGRA profiles. The subjects from this group were characterized by an IFN-γ response to HBHA evolving from positive/strongly positive to clearly negative for some of them, and/or by an IFN-γ response to rESAT-6 evolving from positive/strongly positive to clearly negative, showing sometimes a reciprocal pattern to the HBHA response. This resulted in variable to highly variable ratios of the HBHA to the rESAT-6 scores. Subjects 13, 14 and 15 had most often ratios <1, sometimes extremely low when no IFN-γ response to HBHA was found, together with strongly positive rESAT-6-IGRA results. This profile may reflect a predisposition to develop TB as a result of *Mtb* reactivation or of re-infection as subjects 13 and 14 were potentially re-exposed to *Mtb*, and as previous reports on TB contacts showed that mainly high responders to rESAT-6 or in the QFT-G-IT have an increased risk of developing TB [25], [32], [33]. Further support for this contention is provided by the case of a chronically HD dialyzed patient. Whereas he had initially a very high response to HBHA and a positive response in the QFT-G-IT, the HBHA-IGRA became negative two years later when the patient had symptoms suggestive of active TB, whereas the QFT-G-IT remained positive, and the rESAT-6-IGRA gave extremely high responses. This change in the HBHA- and rESAT-6-IGRA profile was associated with a TB reactivation as confirmed by the gradual improvement of the patient during the anti-TB treatment and later on by the positive *Mtb* culture. Subjects with persistently very low ratios of HBHA/rESAT-6 IFN-γ responses are probably those who should benefit from IPT especially when exposed to immuno-depressive conditions.

For other subjects in this group with changing profiles (subjects 16 to 19), the ratio between the scores of the HBHA and rESAT-6 IFN-γ responses was only occasionally below 1, e. g. as a result of an important transient rise of the IFN-γ response to rESAT-6. Subjects 16 and 18 were potentially re-exposed to *Mtb*. This was not the case for subjects 17 and 19, so that the transiently positive response to rESAT-6 may have resulted from subclinical and transient reactivation of mycobacterial replication. However, the rapid increase of the ratio in these subjects suggests that the protective immune responses were sufficiently strong to prevent full-blown reactivation. IPT may therefore not be necessary, but it may be advisable to continue monitoring these subjects.

Finally the last group of four individuals was characterized by a progressive disappearance of the IFN-γ responses to the mycobacterial antigens, including to PPD for two of them. This kinetic profile is consistent with clearance of the mycobacterial infection. Interestingly, three subjects had undergone IPT, whereas the remaining one had remained untreated. Nevertheless, this latter individual evolved within a period of approximately ten years from a strongly positive to a totally negative HBHA responder, and became finally negative for all tested antigens. Of note, none of the subjects from this group had a positive QFT-G-IT test compared to 5/7 positive in the group with variable ratios and 4/12 in the group with stable ratios. However, the QFT-G-IT and the rESAT-6-IGRA results, when available, together did not always match. This difference could be due to the presence of additional antigens in the QFT-G-IT or to technical differences between the two tests.

In conclusion, kinetic profiling of HBHA-IGRAs and rESAT-6-IGRAs allowed us to subdivide the LTBI population of a low TB incidence country into three groups and to propose a model which may represent the LTBI spectrum (Figure 6) [10]. Using HBHA- and rESAT-6-IGRA applied to the LTBI cohort studied here, we propose that a small proportion (four out of 23, group C) cleared the infection, whereas most (roughly 50%, group A) featured a stable HBHA- and rESAT-6-IGRA profile, with a strong HBHA-specific IFN-γ response and are most likely protected from disease. Roughly 30% (group B) had variable rESAT-6-IGRA responses with consistently high or variable HBHA responses, resulting in fluctuation of the HBHA/rESAT-6 ratios from high to values below 1 and vice-versa. We suggest that group A and group C do not require further attention, whereas group B should be closely monitored as it may comprise individuals at risk for reactivation that should preferentially benefit from IPT. Further refinement of the identification of the individuals at risk to develop active TB will perhaps be possible by combining to IFN-γ the measurement of other cytokines [34] and further studies should help to define how many measurements would be necessary for the definite TB re-activation risk stratification. However, this study represents thus a first attempt to stratify LTBI subjects in different risk-associated groups with proposed indications of IPT.
